# Null Mutations in EphB Receptors Decrease Sharpness of Frequency Tuning in Primary Auditory Cortex

**DOI:** 10.1371/journal.pone.0026192

**Published:** 2011-10-12

**Authors:** Irakli Intskirveli, Raju Metherate, Karina S. Cramer

**Affiliations:** Department of Neurobiology and Behavior and Center for Hearing Research, University of California Irvine, Irvine, California, United States of America; University of Auckland, New Zealand

## Abstract

Primary auditory cortex (A1) exhibits a tonotopic representation of characteristic frequency (CF). The receptive field properties of A1 neurons emerge from a combination of thalamic inputs and intracortical connections. However, the mechanisms that guide growth of these inputs during development and shape receptive field properties remain largely unknown. We previously showed that Eph family proteins help establish tonotopy in the auditory brainstem. Moreover, other studies have shown that these proteins shape topography in visual and somatosensory cortices. Here, we examined the contribution of Eph proteins to cortical organization of CF, response thresholds and sharpness of frequency tuning. We examined mice with null mutations in EphB2 and EphB3, as these mice show significant changes in auditory brainstem connectivity. We mapped A1 using local field potential recordings in adult *EphB2* ^−/^^−^;*EphB3* ^−/^^−^ and *EphB3* ^−/^^−^ mice, and in a central A1 location inserted a 16-channel probe to measure tone-evoked current-source density (CSD) profiles. Based on the shortest-latency current sink in the middle layers, which reflects putative thalamocortical input, we determined frequency receptive fields and sharpness of tuning (Q_20_) for each recording site. While both mutant mouse lines demonstrated increasing CF values from posterior to anterior A1 similar to wild type mice, we found that the double mutant mice had significantly lower Q_20_ values than either *EphB3* ^−/^^−^ mice or wild type mice, indicating broader tuning. In addition, we found that the double mutants had significantly higher CF thresholds and longer onset latency at threshold than mice with wild type *EphB2*. These results demonstrate that EphB receptors influence auditory cortical responses, and suggest that EphB signaling has multiple functions in auditory system development.

## Introduction

Central auditory pathways display tonotopic organization that originates in the cochlea, where receptor cells are ordered by frequency selectivity [1]. Tonotopy is preserved throughout the auditory pathway through topographically ordered neuronal projections and is reflected in the arrangement of best frequencies in the auditory cortex [2], [3]. The development of tonotopic projections requires both activity-independent mechanisms and subsequent activity-dependent refinement [4]. Initial tonotopic targeting is regulated by axon guidance molecules. Signaling through Eph receptors and their corresponding ephrin ligands is required for formation of tonotopy in the auditory brainstem [5], [6] and in the auditory midbrain [7]. In this study we tested the role of Eph receptors in the formation of tonotopy and frequency tuning in the primary auditory cortex.

Eph receptors are the largest family of receptor tyrosine kinases. They are subdivided according to sequence homology into the EphA and EphB classes [8], which bind to ephrin-A's and ephrin-B's respectively, displaying promiscuity within each class [9], [10]. Exceptions to this class specificity have been found, as EphA4 binds to ephrin-B ligands [10] and EphB2 binds to ephrin-A5 [11]. Ephrins are associated with cell membranes by a GPI linkage for the ephrin-A's and through a transmembrane domain for ephrin-B's. The membrane association of both ligand and receptor facilitates cell-cell interactions that result in bidirectional signaling. Both forward signaling through Eph receptors and reverse signaling through ephrins have been shown to be important in nervous system development [12].

Both EphA and EphB signaling pathways have been shown to play a role in establishing topographic projections during development in several different areas of the nervous system. The best documented pathway is the retinotectal projection, where graded levels of expression along the axes of topography, together with either attractive or repulsive signaling, have been shown to be necessary for map formation [13], [14], [15], [16]. Ephrin-A signaling is also needed for thalamocortical mapping [17]. Topographic ordering in thalamocortical visual projections along the azimuthal axis has also been shown to require ephrin-A's [18], [19], which work together with spontaneous activity to shape retinotopic maps. Ephrin-A signaling may also contribute to topography in the somatosensory cortex [20], [21] and in areal thalamocortical mapping [22]. The role of EphB signaling is not known for these cortical projections, and the molecular signals that establish and refine tonotopic mapping in the auditory pathway have not been identified.

We previously reported that the compound null mutant *EphB2* ^−/−^;*EphB3* ^−/−^ mice show significant numbers of aberrant projections from the ventral cochlear nucleus (VCN) to the ipsilateral medial nucleus of the trapezoid body (MNTB), whereas in wild type mice VCN projects to contralateral, but not ipsilateral, MNTB [23]. Here we have examined how auditory cortical responses are affected in these mice. Expression studies (Allen Developing Mouse Brain Atlas [Internet]. Seattle (WA): Allen Institute for Brain Science. ©2009. Available from: http://developingmouse.brain-map.org) demonstrated that EphB2 is strongly expressed in the developing auditory thalamus at postnatal day 4 (P4), and in cortex at later ages (P56). This database shows that EphB3 expression is minimal in these areas at both early and late postnatal ages, with low levels of expression at P14 and P28. We used a reporter mouse line to obtain more detailed EphB2 expression data. We found that EphB2 is expressed in the early postnatal auditory thalamus, and that expression in the auditory cortex is seen after hearing is mature. In order to determine how these mutations influence auditory cortical responses, we mapped the characteristic frequency (CF), the frequency that elicits a response at the lowest intensity, along the tonotopic axis of A1. We then determined the sharpness of tuning at one site, using CSD analysis to infer the cortical response to thalamic input. Our results show that the mutant mice have normal tonotopic ordering for CF in A1, but that the sharpness of frequency tuning is significantly lower in mutants than in wild type mice. These studies suggest that Eph receptors are needed for precision in neuronal connectivity at multiple points in the central auditory system.

## Materials and Methods

### Animals

For electrophysiological studies we used 56–70 day old male mice from a line of mice containing null mutations in *EphB2* and *EphB3* [24], [25] on a CD-1/129 background (WT n = 4; *EphB2* ^−/−^;*EphB3* ^−/−^ n = 4; and *EphB2* ^+/+^;*EphB3* ^−/−^ n = 5). For expression studies we used mice heterozygous for *EphB2* ^lacZ^, a mutant allele that generates a fusion protein in which the intracellular domain of EphB2 is replaced with β-galactosidase [24], [25]. For these studies we used animals at postnatal day 4 (P4; n = 3) and P60 (n = 3). We used PCR genotyping as previously described [23]. All procedures were approved by the University of California, Irvine Institutional Animal Care and Use Committee (protocol 2002–2389) and were performed in accordance with the NIH Guide for the Care and Use of Laboratory Animals.

### β-Galactosidase Histochemistry

Mice were anesthetized with inhaled Isoflurane (Abbott Laboratories) then perfused transcardially with 0.9% NaCl then 4% paraformaldehyde (PFA) in 0.1 M phosphate buffered saline (PBS; pH 7.4). Brains were fixed in PFA for 15 minutes then equilibrated overnight in 30% sucrose in phosphate buffered saline. The thalamus and cortex were cryosectioned in the coronal plane at 14 µm. Sections were rinsed in PBS then incubated at 37°C in an X-gal staining solution containing 5 mM ferrocyanide, 5 mM ferricyanide, 2 mM MgCl, and 1 mg/ml 5-bromo-4-chloro- 3-inolyl,betaD-galactopyranoside (X-gal; Sigma, St. Louis, MO) in PBS for several hours. Slides were then rinsed in PBS then coverslipped with Glycergel mounting medium (Dako, Carpinteria, CA). A series of alternate sections was stained with thionin to aid in identifying brain regions.

### In-vivo electrophysiology

Electrophysiological recordings and analysis were performed blind to genotype. Mice were anesthetized with urethane (Sigma; 0.7 g/kg i.p.) and xylazine (Phoenix Pharmaceutics; 13 mg/kg i.p.) in saline, placed in a sound-attenuating chamber (model AC-3, IAC, Bronx, NY) and maintained at 36–37°C. Anesthesia was supplemented as necessary (0.13 g/kg urethane, 13 mg/kg xylazine i.p.) via a catheter to avoid movement of the mice. The head was secured in a stereotaxic frame (model 923, Kopf Instruments, Tujunga, CA). After a midline incision the skull was cleared and secured using a custom made head holder. A craniotomy was performed over the right auditory cortex and the exposed brain was kept moist with warm saline.

Tone-evoked local field potentials (LFPs) were obtained using a glass microelectrode (1 M NaCl, ∼1 MΩ at 1 kHz,) or a 16-channel silicon multiprobe (100 µm separation between each 177 µm^2^ recording site, 2–3 MΩ at 1 kHz; NeuroNexus, Ann Arbor, MI, USA) and were filtered and amplified (1 Hz–10 kHz, AI-401 or AI-405 CyberAmp380), digitalized (AxoGraph) and stored on a Macintosh computer (Apple Computer).

To find a recording site in A1, we modified our method previously described for rat [26]. Initially, we recorded tone evoked LFPs from multiple sites (distance between sites ∼250 µm) along the rostrocaudal axis in auditory cortex. At each site, we presented tones ranging in frequency from 3 kHz to 40 kHz in 2.5 kHz steps, and ranging in intensity from −10 dB to 70 dB sound pressure level (SPL). CF was determined at each recording site from the superficial layers (<100 µm from surface), and for a few sites in each animal, replicated by recording multiunit activity in layer IV (∼400 µm depth).

To identify A1, we confirmed the expected tonotopic gradient of CF including the reversal of tonotopy indicating the border with the anterior auditory field. We then selected a recording site within A1 by mapping along the dorsal-ventral axis and determining the site with the shortest-latency and largest-amplitude surface LFP response to 10−70 dB tones. Once a site was selected, we inserted the multiprobe perpendicular to the pial surface and re-determined CF with greater precision by measuring the initial slope and onset latency of LFPs in layer IV, 300−400 µm from the pial surface. CF (1 kHz steps) elicited the shortest-latency LFP response at threshold (5 dB steps; defined as the lowest intensity to evoke an LFP above baseline (>3 s.d. above 100 ms pre-tone average).

### Acoustic stimuli

Acoustic stimuli were digitally synthesized and controlled using MALab (Kaiser Instruments, Irvine, CA) and dedicated computer (Macintosh PowerPC, Apple Computer) and delivered through a speaker (ES-1 with ED-1driver, Tucker-Davis Technologies, FL) positioned ∼3 cm in front of the left ear. For calibration (SPL in dB re: 20 µPa) a microphone (model 4939 microphone and Nexus amplifier; Brüel and Kjaer, GA) was positioned in the place of the animal at the tip of the left ear-bar. Tones were 100 ms in duration with 5 ms linear rise and fall ramps.

### In-vivo data analyses

We averaged LFP responses to sets of 25 stimuli and carried out CSD analysis [26], [27]. The onset latency of the middle layer current sink was defined at the first of consecutive data points, at least 3 ms duration, that was above a threshold amplitude (2 s.d. above the mean 10 ms baseline preceding tone onset). The current sink in the middle layers with the shortest latency was considered the location of thalamocortical input, and is assumed to reside near the layer III-IV border. After determining the current sink's frequency vs. intensity response area, to quantify sharpness of tuning, we measured bandwidth 20 dB above threshold and determined Q_20_:


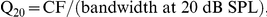


where greater values indicate sharper tuning. We compared mean Q_20_ values between genotype groups.

## Results

### EphB2 expression

**Figure 1:**
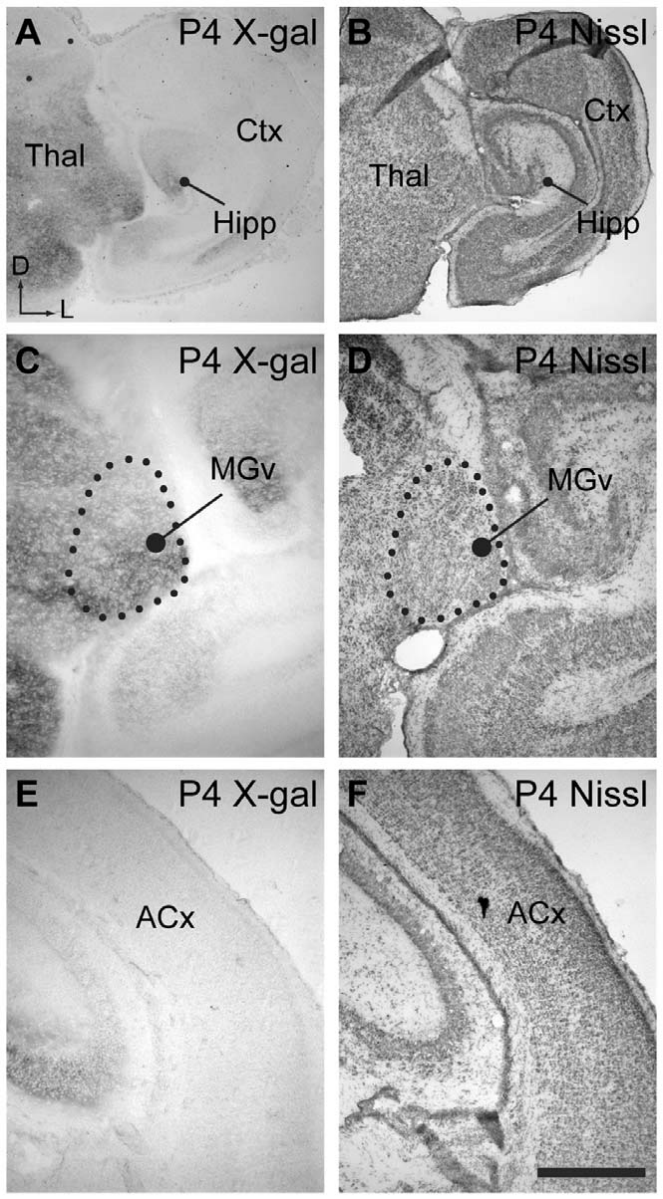
Expression of EphB2 in auditory pathways at P4. **A**. Low power view of coronal P4 brain section from an EphB2^lacZ/+^ mouse showing X-gal histochemistry. Reaction product is abundant in the thalamus (Thal), and labeling is seen in the hippocampus (Hipp). Cortical regions (Ctx) show relatively little labeling. Axes: D, dorsal and L, lateral; applies to all panels. **B**. Section adjacent to that shown in A with Nissl staining to highlight brain regions. **C**. Higher power view of coronal section through thalamus in EphB2^lacZ/+^ mouse processed for X-gal histochemistry. Labeling is evident within the ventral medial geniculate nucleus (MGv). **D**. Nissl staining in section adjacent to that shown in A, used to identify thalamic regions. **E**. Coronal section showing region of auditory cortex (ACx), with minimal X-gal labeling. **F**. Adjacent section to that shown in C, with Nissl staining. Scale bar in F represents 500 µm for panels A−B and 200 µm for panels C−F.

Data from the Allen Developing Mouse Brain Atlas showed strong expression of EphB2 (but not EphB3) in the developing thalamus. We performed expression studies to obtain more detailed expression analysis in coronal sections, which reveal subdivisions of the medial geniculate nucleus. We used lacZ reporter mice to investigate the expression of EphB2 in the mouse auditory thalamus and cortex at P4 (n = 3), prior to hearing onset, and at P56-60 (n = 3), when hearing is mature and when electrophysiological recordings were made. Figure 1 shows the expression pattern revealed by X-gal labeling together with adjacent thionin labeled coronal sections for P4 animals, used to determine anatomical regions. The ventral division of the medial geniculate body (MGv), which projects tonotopically to A1, shows strong labeling in P4 tissue (Fig. 1A−D). In contrast, the dorsal division (MGd) was less intensely labeled at this age. Labeling in the auditory cortex was not observed in P4 mice (Fig. 1E,F).

**Figure 2:**
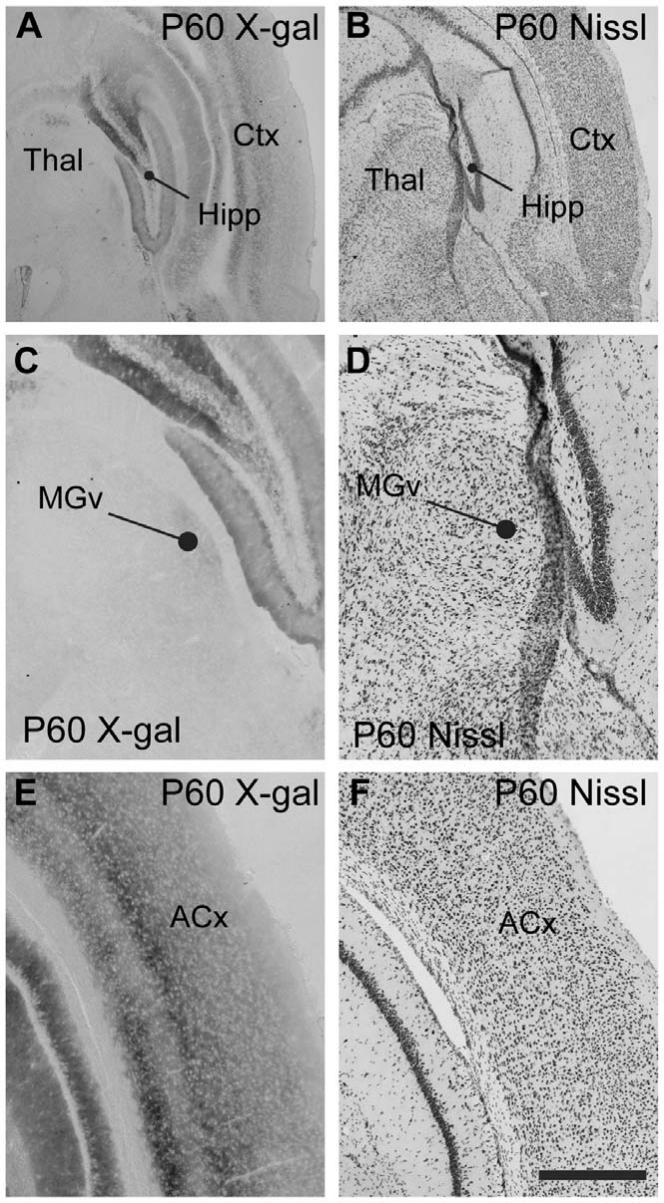
Expression of EphB2 in auditory pathways at P60. **A**. Coronal section through P60 EphB2^lacZ/+^ mouse brain, showing reaction product in hippocampus (Hipp) and cerebral cortex (Ctx), with relatively little labeling in the thalamus (Thal). **B**. Nissl stained section adjacent to that shown in A. **C**. Coronal section processed for X-gal histochemistry showing thalamus in EphB2^lacZ/+^ mouse. The ventral medial geniculate nucleus (MGv) shows very low levels of labeling. **D**. Nissl stained section adjacent to that shown in A. **E**. X-gal labeling is seen throughout cortex at this age, and includes auditory regions (ACx). **F**. Nissl stained section adjacent section to that shown in C. Axes for all panels as in Figure 1. Scale bar in F, 500 µm for panels A and B; 200 µm for panels C−F.

The expression patterns of EphB2 change dramatically during development (Fig. 2A,B). At P60, the auditory thalamus lacks reporter expression (Fig. 2C,D), while labeling is present throughout the cortex, including the auditory cortex (Fig. 2E,F). These results are consistent with the Allen Developing Mouse Brain Atlas, and in addition, the reporter mice show the expected expression patterns in other brain regions (Fig. 2A,B), including the hippocampus [28].

### Electrophysiology

#### Tonotopic organization of auditory cortex in wild type and mutant mice

**Figure 3:**
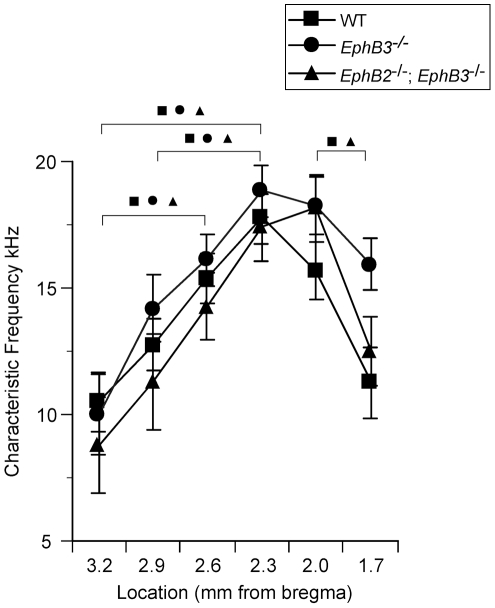
Tonotopic progression of CF is similar in mutant and wild type auditory cortex. For all animals, CF was determined at recording sites separated by ∼300 µm along the anterior-posterior axis of cortex. Mean CF for each group is plotted according to position relative to bregma (all sites ∼6 mm lateral to midline). Symbols indicate significant differences (ANOVA with post-hoc paired t-tests, p<0.05) between recording sites within the same mouse strain, indicating topographic progression of CFs and reversal of topography between presumed A1 and the anterior auditory field.

The spatial organization of CF across the caudal-to-rostral axis of A1 was obtained by recording tone-evoked LFPs using a single glass electrode in 13 mice that represented three different genotypes (WT, n = 4; *EphB2* ^−/−^; *EphB3* ^−/−^ n = 4; *EphB3* ^−*/*−^ n = 5). Recordings targeted superficial (<100 µm below pia) or middle (400 µm) cortical layers, but CF determined at either depth was similar (within ∼1 kHz). The initial surface recording site in each animal was located 2.0 mm posterior to bregma and ∼6 mm lateral to the midline at a location expected (by experience) to be in either anterior A1 or the posterior portion of the anterior auditory field. Almost invariably, CF at this location was ∼15–20 kHz. Subsequent recordings were made in ∼300 µm steps in both posterior and anterior directions to determine the tonotopic progression of CFs and the location of the tonotopy reversal indicating the border between A1 and the anterior field. We observed similar tonotopic organization for A1 in all genotypes (Fig. 3), with CFs at the caudalmost recording sites being lower frequency (7.5–10.5 kHz) and increasing gradually along the posterior-to-anterior axis until high frequencies (∼20 kHz) were observed, followed by a reversal of tonotopy (Fig. 3). Note that higher-resolution mapping would likely reveal higher-frequency CFs near the border of A1 and the anterior auditory field. All genotypes showed an increase in CF with steps in the anterior direction followed by a reversal of tonotopy that indicates the boundary of A1 with the anterior auditory field (for each group, ANOVA followed by post-hoc paired t-tests with outcome indicated by symbols in Fig. 3: wild type, p = 0.0027, n = 4; EphB3-/-: p = 0.009, n = 5; EphB2-/-; EphB3-/: p = 0.0062). At each location along the anterior-posterior axis, CF did not differ among phenotypes (ANOVAs, n = 3−5, p>0.05).

#### Functional connectivity measured using CSD analysis

**Figure 4:**
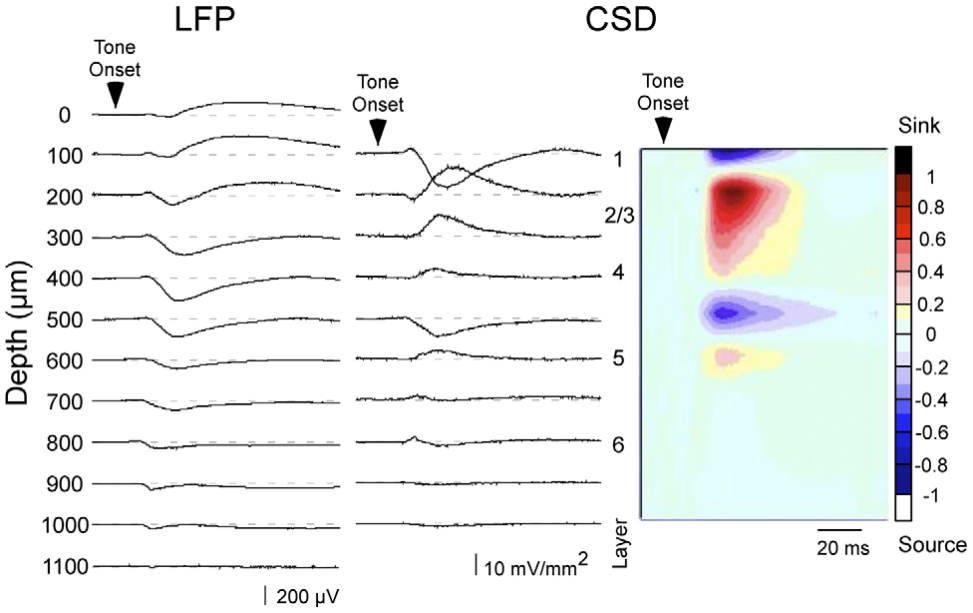
Laminar profile of CF tone-evoked responses in A1 of wild type mouse. A representative example of LFPs (left column), derived one-dimensional CSD traces (middle) and interpolated color plot (right) shows the response to a CF stimulus. Responses shown are from the top 12 recording sites of a 16-channel silicon multiprobe, which span the entire cortical thickness; the most superficial site (labeled 0 µm) was visible at the cortical surface. Color scale indicates response amplitude normalized to the largest current sink (reds/black) and largest current source (blues/white), and also applies to all subsequent CSD figures.

Once we determined the location of A1 by mapping CF, we studied individual sites in more detail. A 16-channel silicon multiprobe was inserted orthogonal to the cortical surface in a region clearly within A1 (CF<20 kHz), to record tone-evoked LFPs simultaneously from all cortical layers (100 µm separation between recording sites) in *EphB2* ^−/−^; *EphB3* ^−/−^, *EphB2* ^+/+^; *EphB3* ^−/−^ and wild type mice. Figure 4 shows an example of tone-evoked LFPs and the derived CSD profile elicited in response to CF stimuli in a wild type mouse. Typical CSD profiles exhibited one or two current sinks in the middle to upper layers (200–400 µm depth): within this range, the current sink with the shortest-latency onset most likely reflects thalamocortical input. This shortest-latency current sink typically had a smaller peak amplitude than the longer-latency and larger (main) current sinks located more superficially, ∼200–300 µm below the pial surface. The middle layer current sinks were bounded by current sources in layers I and V. In deeper layers, a short-latency, small-amplitude current sink was observed often (600 µm depth in Fig. 4), and may reflect inputs from thalamocortical collaterals.

**Figure 5:**
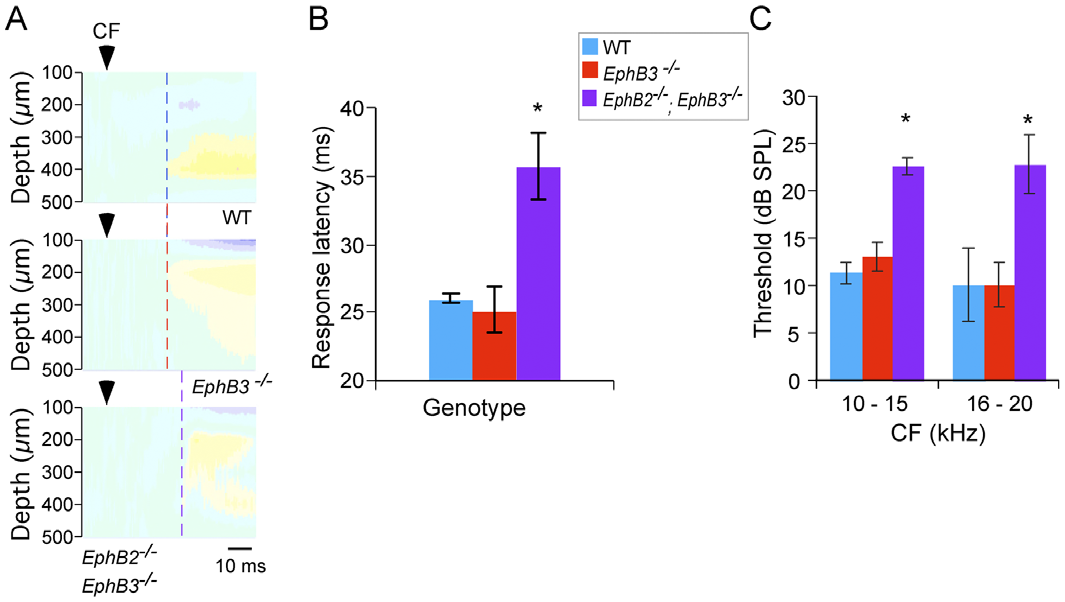
CF-evoked, layer IV current sinks in double mutant *EphB2* ^−^ ^***/*−**^ ***; EphB3*** **^−^** ^***/*−**^ **mice have longer onset latencies and higher thresholds.** **A**. Representative CSD profiles in wild type, single- and double-mutant mice show onset latency (vertical dashed line) of earliest middle-layer current sink in response to threshold-intensity CF stimulus (arrowhead indicates tone onset). Response latency was similar in wild type and *EphB3* ^−*/*−^ mice, and longer in *EphB2* ^−*/*−^ *; EphB3* ^−*/*−^ mouse. **B**. Mean (± SEM) onset latency to threshold stimulus was longer in double-mutant mice (*, post-hoc tests, p<0.05). **C**. Mean CF threshold was higher in double-mutant mice (*, post-hoc tests, p<0.05). For across-strain comparisons, recording sites with similar CFs were grouped.

CF tone-evoked CSD profiles in mutant mice were qualitatively similar to those in WT mice, with the same profile of alternating current sinks and sources across layers I - VI. Within this general pattern, variability in CSD profiles fall within the normal range [29], [30], and are not attributed to genetic mutations. Quantitative analyses of the layer IV (putative thalamocortical) current sink showed that *EphB2* ^−/−^; *EphB3* ^−/−^ mice had significantly longer response onset latencies at CF threshold than *EphB3* ^−*/*−^ and WT mice (Fig. 5A, B) (WT 25.91±0.35 ms; *EphB3* ^−*/*−^ 25.14±1.65 ms; *EphB2* ^−/−^; *EphB3* ^−/−^ 35.65±2.48 ms; p<0.005, ANOVA with post-hoc Tukey HSD test). Moreover, an analysis of CF threshold revealed that *EphB2* ^−/−^; *EphB3* ^−/−^ mice had higher thresholds than did *EphB3* ^−*/*−^ and WT mice (Fig 5C; for CF 10–15 kHz: WT 11±1.17 dB; *EphB3-/-* 13±1.48 dB; *EphB2*-/-; *EphB3*-/- 22.5±0.96 dB, p<0.0001, ANOVA with post-hoc Tukey HSD test; for CF 16-20 kHz: WT 10±3.89 dB; *EphB3-/-* 10±2.36 dB; *EphB2*-/-; *EphB3*-/- 22.7±3.11 dB; p<0.01, ANOVA with post-hoc Tukey HSD test). However, the amplitude of the layer IV current sink, either at threshold or 20 dB above threshold, did not differ among groups (at threshold: WT 7.4±2.4 mV/mm^2^; B3^−**/**−^ 7.96315±2.1971 mV/mm^2^; B2^−/−^; B3^−/−^ 2.20431±2.4564 mV/mm^2^; ANOVA, p>0.05; at 20 dB above threshold: WT 12.512±6.4052 mV/mm^2^; B3^−**/**−^ 19.0423±5.729 mV/mm^2^; B2^−/−^; B3^−/−^ 5.6168±6.4052 mV/mm^2^; ANOVA, p>0.05). Note that since onset latency and threshold vary with CF, only recording sites with CF of 10–20 kHz (the large majority of recording sites) were included in across-strain comparisons.

**Figure 6:**
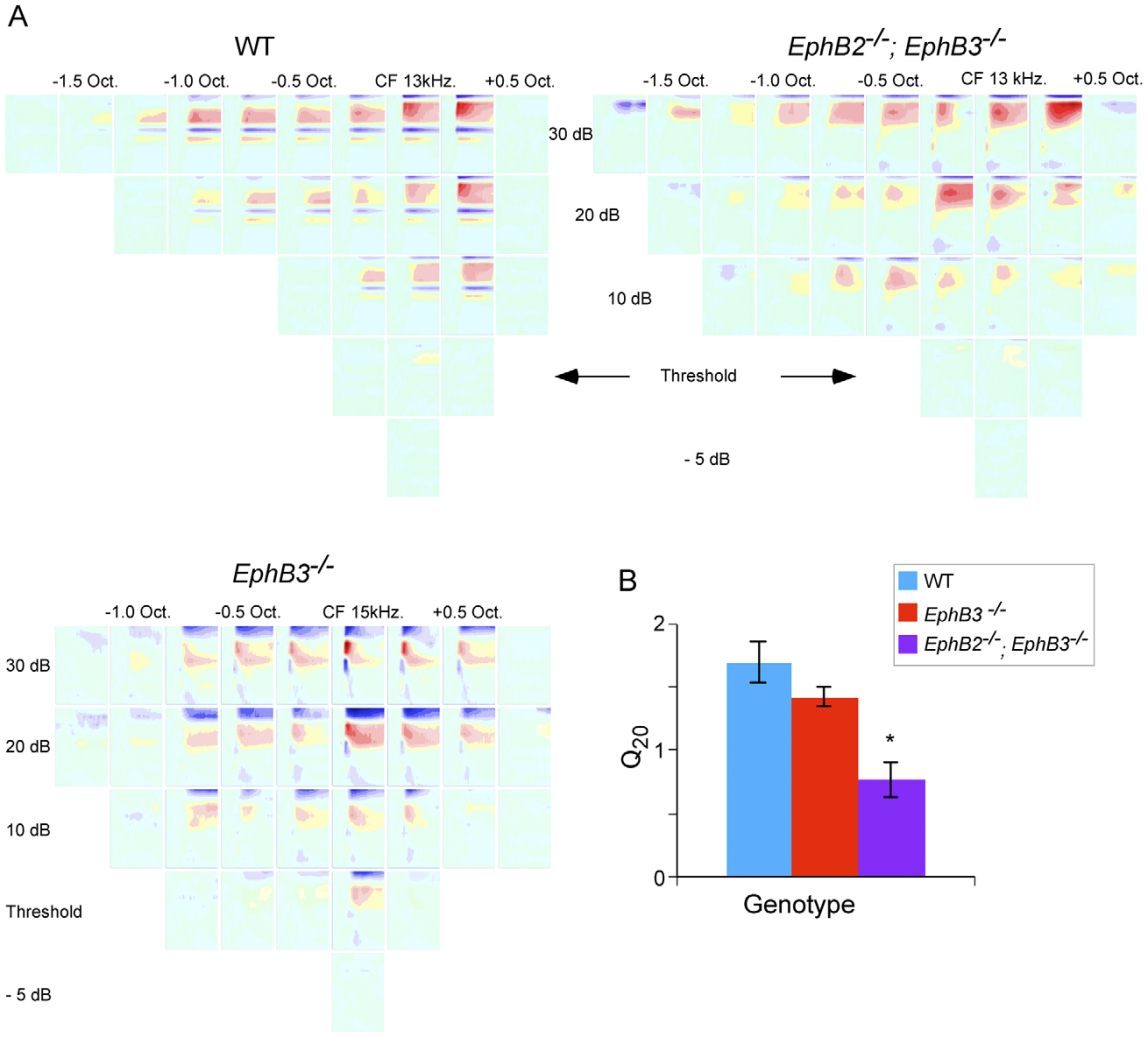
Frequency receptive fields derived from layer IV current sink are broader in double mutant, *EphB2* ^−^ ^***/*−**^ ***; EphB3*** **^−^** ^***/*−**^ **mice.** A, Representative CSD profiles in A1 of wild type (top left), *EphB2* ^−*/*−^ *; EphB3* ^−*/*−^ (top right) and *EphB3* ^−*/*−^ (bottom) mice, obtained in response to tones of frequencies ranging from 1.75 octaves below CF to 0.5 octaves above, and intensities ranging from 5 dB below threshold to 30 dB above. Illustrated examples have thresholds at CF of 15 dB (top left), 20 dB (top right) and 20 dB (bottom) SPL. B, Mean (± SEM) bandwidth of layer IV current sink measured at 20 dB above CF threshold (Q_20_). *, post-hoc tests, p<0.005

Finally, we compared frequency receptive fields in mutant and wild type mice. Receptive fields were determined using the peak amplitude of the layer IV current sink, since it reflects an early cortical response to thalamic input. We determined the response of the layer IV sink to a range of frequencies (1–40 kHz in 0.25 octave steps) and intensities (from below threshold to 70 dB in 5 or 10 dB steps). Fig. 6A shows representative receptive fields from a wild type mouse (top left), an *EphB2* ^−/−^; *EphB3* ^−/−^ mouse (top right) and an *EphB3* ^−*/*−^ mouse (bottom). For quantitative analysis, we determined receptive field breadth 20 dB above threshold (Q_20_, Fig. 6B), which showed that receptive fields of double mutant *EphB2* ^−/−^; *EphB3* ^−/−^ mice (Q_20_ = 0.76±0.13) were more broadly tuned than those of either *EphB3* ^−*/*−^ (Q_20_ = 1.42±0.07) or wild type mice (Q_20_ = 1.68±0.16; p<0.005, ANOVA with post-hoc Tukey HSD test).

## Discussion

Eph receptor tyrosine kinases and their ligands have important roles in the establishment of the precise connectivity observed in the auditory brainstem. In this study we investigated whether they are also important for establishing auditory cortical organization. While both EphA and EphB receptors have been shown to guide formation of topographic maps, we focused this initial study on EphB receptors because of their extensive expression in the auditory system and their role in establishing precision in auditory brainstem pathways. We showed that EphB2 is highly expressed in the auditory thalamus during early development, but not in mature animals. In contrast, older animals lack EphB2 expression in the auditory thalamus but show extensive expression throughout sensory cortex. In mice lacking EphB2 and EphB3, we observed normal tonotopic ordering of CF in A1, but when we assessed early cortical responses to acoustic inputs we found that frequency selectivity was significantly decreased compared to wild type mice and mice with mutations lacking only EphB3. In addition, the double mutant mice had significantly longer latencies and elevated thresholds. These results provide the first demonstration that Eph proteins influence the organization of inputs to the auditory cortex.

Eph proteins have several known functions and may act at multiple points along the auditory pathway to account for the effects we observed in the mutant mice. Given the high levels of expression of EphB2 in the MG at early ages, a possible explanation is that Eph signaling provides axon guidance cues that restrict auditory thalamocortical axons to appropriate tonotopic locations. While EphB3 is not expressed in the thalamus at early postnatal ages, it may nonetheless influence this pathway, as expression is seen in cortex and at low levels in thalamus at P14 and P28 (Allen Developing Mouse Brain Atlas, *op*. *cit.*). In the auditory brainstem, EphB2/EphB3 double mutants elicit some phenotypes not seen with either mutation alone. It is thus likely that EphB2 and EphB3 both contribute to the establishment of frequency selectivity in A1. The difference in the timing of expression of these proteins is consistent with distinct functions; for example EphB2 may guide growing thalamocortical axons while both proteins may regulate refinement and stabilization of connections. While the spatial resolution of our recordings may be insufficient to detect the detailed organization of mouse MGv-A1 projections [31], [32], the CSD analysis yields measurements of CF, thresholds, and frequency selectivity that can be attributed to the earliest cortical response to thalamocortical input [26], [33], [34].

Eph receptors and ephrins are required for topographic mapping in the visual cortex [14], [18], [35], [36] and somatosensory cortex [20], [21], [22], and also provide topographic guidance cues for thalamic projections to appropriate areas of cortex [22], [37]. While all of these studies showed a role for the EphA subclass, EphB signaling has been shown to establish topographic mapping in retinotectal projections. Our observation of decreased frequency selectivity in the double mutant mice is consistent with broader divergence in the projections from MG to A1. MG neurons might preserve their neighbor relationships in their projection to A1, but their terminations may extend more broadly across the frequency axis. Each region of A1 would thus receive inputs from MG that represent a greater range of frequencies. This type of broadening of topographic projections has been demonstrated anatomically in Eph mutant mice in visual pathways [38], [39]. In addition, misexpression of EphA4 resulted in broadening of auditory brainstem connections during embryonic development in chicks [5].

An additional possibility is that EphB receptors are needed for tonotopic ordering at lower levels of the auditory pathway, such as in projections to the inferior colliculus, or to MG. In this case, targeting errors in the mutant mice could be relayed to A1 via normally mapped thalamocortical afferents. While our study did not directly address this possibility, it is unlikely to account entirely for the effects we observed, as our data suggest the mutations result in abnormal inputs to layer IV. The increase in thresholds in mutants could result from weaker synaptic drive in any part of the ascending auditory pathway. This analysis suggests that EphB signaling has effects on multiple projections throughout the auditory pathway. While a role for intrinsic and descending connections cannot be ruled out, the data are consistent with defects in the ascending pathway.

An important factor in the development of topographic maps is the role of activity-dependent refinement, which results in narrowing of termination zones and elimination of less appropriate inputs. Eph proteins act together with spontaneous activity to establish retinotopic maps [39]. In the auditory brainstem, the topographic projection from the MNTB to the lateral superior olive (LSO) undergoes activity-dependent refinement. In the early postnatal period, LSO neurons receive large number of small inputs. Synapse elimination and strengthening of remaining synapses leads to a more refined tonotopic projection with stronger synaptic weights [4], [40]. Refinement of tonotopy in auditory cortex also occurs by activity-dependent mechanisms. Exposure to white noise in the postnatal period prevents refinement in rat A1 [41]. Activity-dependent maturation of cortical tonotopy may require acetylcholine neurotransmission, as A1 neurons in mice lacking M1 muscarinic ACh receptors have poor frequency selectivity and elevated thresholds [42].

In the EphB2/EphB3 double mutants, activity dependent refinement did not adequately correct for any mapping defects. An interesting possibility is that in mutant mice, elevated thresholds may also reflect a failure to strengthen synapses during development. In addition to their role in axon guidance, EphB receptors have a well-documented role in synapse maturation [43], [44], [45], [46]. Reduction in the activity of these proteins in mutants may delay or impair the adjustment of synaptic input strength. Thus Eph proteins may regulate auditory cortical function through multiple signaling functions.

EphB signaling in axon guidance and synapse formation could influence the auditory pathway at many points. While our study focused on auditory cortical responses, analysis of physiological and anatomical connections throughout the auditory system of mutants would be needed to determine which aspects of the circuitry are affected. This approach will provide an improved understanding of the developmental factors that contribute critically to auditory circuit assembly and function.

The role of Eph proteins in topographic mapping has been determined in many pathways, and has been best characterized in the visual pathway. The present study extends this role for Eph signaling to the auditory cortex. In the visual pathway, opposing expression gradients of EphA receptors along the temporonasal axis of retinal ganglion cell axons and ephrin-A ligands along the anteroposterior axis in the tectum facilitate chemorepulsive interactions that shape retinotectal mapping. Interestingly, these EphA proteins use a similar strategy to promote topographic ordering in several projections along the visual hierarchy, including the geniculocortical pathways where they seem to selectively influence temporonasal mapping [18]. Gradients of EphB proteins establish topography along the dorsoventral axis in retinotectal projections, but their signaling appears more complex. EphB signaling regulates interstitial branching, and signals are attractive or repulsive depending on concentration of protein molecules [14], [47]. The role of EphB signaling in the formation of visual thalamocortical maps has not been established, but it remains possible that they play a role in mapping along the dorsoventral axis and in regulating branching in the cortex as well.

The early auditory pathways rely on both EphA and EphB signaling to establish appropriate connections. Signaling mechanisms likely differ somewhat from the visual pathway, as we have observed graded expression of some Eph proteins, but have not detected opposing gradients of ligands. Additionally, there appears to be greater variability in Eph family members that contribute to axon pathfinding in auditory areas, as mutations in EphA4 and ephrin-B2 result in tonotopic defects in distinct brainstem nuclei [6]. These differences may be accounted for in large part because the brainstem circuitry in auditory pathways is not comparable to early visual pathways. Additionally, our studies have focused on mapping along a single axis, the frequency axis, whereas visual system studies reveal distinct mechanisms for mapping along two orthogonal axes. Thus, comparison of more similar structures, such as the thalamocortical pathways, will be instructive. While projections to different primary cortical areas share many features, these areas may have distinct intrinsic molecular determinants [48], [49], [50]. Taken together, work in this area demonstrates that Eph proteins are extensively used in a variety of ways to orchestrate formation of neural circuits. The diverse family members, signaling mechanisms, and functions contribute to the highly ordered and precise connections needed for cortical function.
